# Relationship between Self-Reported Dietary Nutrient Intake and Self-Reported Sleep Duration among Japanese Adults

**DOI:** 10.3390/nu9020134

**Published:** 2017-02-13

**Authors:** Yoko Komada, Hajime Narisawa, Fumitaka Ueda, Hitomi Saito, Hiroyuki Sakaguchi, Makoto Mitarai, Rina Suzuki, Norihisa Tamura, Shigeru Inoue, Yuichi Inoue

**Affiliations:** 1 Department of Somnology, Tokyo Medical University, 6-1-1 Shinjuku, Shinjuku-ku, Tokyo 160-8402, Japan; ykoma@tokyo-med.ac.jp (Y.K.); haji.nari@gmail.com (H.N.); tamura65@tokyo-med.ac.jp (N.T.); 2 Japan Somnology Center, Neuropsychiatric Research Institute, 1-24-10 Yoyogi, Shibuya-ku, Tokyo 151-0053, Japan; 3 Pharmaceutical and Healthcare Research Laboratories, FUJIFILM Corporation, 2-5-1 Suwa, Tama, Tokyo 206-0024, Japan; fumitaka.ueda@fujifilm.com (F.U.); hitomi.saito@fujifilm.com (H.S.); hiroyuki.sakaguchi@fujifilm.com (H.S.); 4 Marketing Department, Maruha Nichiro Corporation, 3-2-20 Toyosu, Koto-ku, Tokyo 135-8608, Japan; m-mitarai@maruha-nichiro.co.jp (M.M.); ri-suzuki@maruha-nichiro.co.jp (R.S.); 5 Department of Preventive Medicine and Public Health, Tokyo Medical University, 6-1-1 Shinjuku, Shinjuku-ku, Tokyo 160-8402, Japan; inoue@tokyo-med.ac.jp

**Keywords:** sleep duration, midpoint of sleep, dietary nutrients, nutrition, food

## Abstract

Several studies have reported that short sleep duration is a risk factor for obesity and metabolic disease. Moreover, both sleep duration and sleep timing might independently be associated with dietary nutrient intake. In this study, we investigated the associations between self-reported sleep duration and dietary nutrient intake, with and without adjustments for variations in sleep timing (i.e., the midpoint of sleep). We conducted a questionnaire survey, comprising a validated brief self-administered diet history questionnaire (BDHQ) and the Japanese version of the Pittsburgh Sleep Quality Index (PSQI) among 1902 healthy Japanese adults and found that the dietary intakes of several nutrients correlated with sleep duration among men regardless of adjustment for the midpoint of sleep. Particularly, (1) small but significant correlations were observed between sleep duration and the percentage of energy from protein, regardless of adjustment for the midpoint of sleep; (2) energy-adjusted intakes of sodium, vitamin D, and vitamin B12 also significantly correlated with sleep duration; and (3) intakes of bread, pulses, and fish and shellfish correlated with sleep duration. In contrast, no significant correlations were observed between sleep duration and dietary intakes among women. This study revealed that after controlling for the midpoint of sleep, sleep duration correlated significantly with the dietary intake of specific nutrients and foods in a population of Japanese men.

## 1. Introduction

In recent years, laboratory and epidemiologic evidence has identified short sleep duration as a risk factor for the development of obesity and metabolic disease [1,2,3,4]. Indeed, in humans, sleep duration plays an important role in regulating the levels of leptin and ghrelin, which are among the key modulators of appetite and energy expenditure [1,5]. Several studies have shown associations of repetitive partial sleep deprivation and/or chronic short sleep duration with a significant decrease in leptin levels and an increase in ghrelin levels [4,6,7]. This might be related to an increase in subjective hunger experienced by self-restricted individuals [1].

Several studies have investigated the association between sleep duration and dietary intake. In a NHANES (National Health and Nutrition Examination Survey)-based study, Grandner et al. found that relative to normal sleepers (7–8 h), short sleepers (5–6 h) reported higher intakes of absolute protein, carbohydrate, and total fat but a lower intake of dietary fiber, whereas very short sleepers (<5 h) reported lower intakes of protein, carbohydrates, dietary fiber, and total fats [8]. In an analysis of Chinese adults, individuals with a self-reported short sleep duration (<7 h) had a lower carbohydrate intake and higher fat intake, compared to normal-duration sleepers (7–9 h) [9]. Kant et al. also observed that both short and long sleepers reported receiving lower percentages of energy from protein, compared to normal duration sleepers in the NHANES [10].

Several previous studies have therefore emphasized the relationship between short sleep duration and poor dietary intake. A recent report suggested that in addition to sleep duration, sleep timing exhibited an important correlation with obesity [11]. Individuals with later sleep timing were 1.5 times more likely to be obese than were individuals with an early sleep timing, despite reasonably similar sleep durations [11]. Regarding nutrient intake, two recent studies showed the influence of sleep timing on the dietary intakes of certain nutrients [12,13]. A cross-sectional study of a large representative sample of the Finnish population also revealed that individuals with later sleep timing exhibited less healthy dietary habits, such as lower vitamin and higher fat consumption, compared to individuals with earlier sleep timing [12]. Another study of young Japanese women found that later sleep timing (i.e., later midpoint of sleep) was significantly associated with a lower percentage of energy intake from protein and carbohydrates; a lower energy-adjusted intake of cholesterol, potassium, calcium, magnesium, iron, zinc, vitamin A, vitamin D, thiamin, riboflavin, vitamin B6, and folate; and a higher percentage of energy intake from alcohol and fat [13].

Considering the results of the above-mentioned studies, we hypothesized that both sleep duration and sleep timing are associated with dietary nutrient intake. To test this hypothesis, we conducted a survey of sleep and dietary nutrient intake in a Japanese adult population. Our primary aim was to identify the existence of an association between the self-reported sleep duration and the intakes of specific dietary nutrients, after adjusting for variations in sleep timing.

## 2. Methods

### 2.1. Ethical Approval

This study was approved by the ethics committee of Tokyo Medical University (No. 2586), and written informed consent was obtained from all study participants.

### 2.2. Patient Selection

A total of 2007 healthy individuals, aged between 30 and 69 years, participated in this cross-sectional survey. Participants were recruited via study advertisements run by a Clinical Research Organization. Individuals who met the following criteria were excluded from the present study: previous or current diagnosis of psychiatric disorders, history of neurologic illness, use of medication with known effects on sleep or daytime alertness, or shift worker. We also excluded those with self-reported extremely low or high energy intake (<725 or >3235 kcal/day) (*n* = 92), and those whose midpoint of sleep was outside the range of midnight to noon (*n* = 15). After these exclusions, the final sample included in the subsequent analyses comprised 1902 adults.

### 2.3. Demographic Variables

The participants filled out self-administered questionnaires containing questions related to demographics and lifestyle. The demographic items included age, sex, height, weight, current smoking habits (yes or no), current exercise routine (≥2 times/week, ≥30 min/session; yes or no), family structure (living alone or living with other family members).

### 2.4. Assessment of Dietary Intake

Dietary intake during the preceding one month was assessed with a validated, self-administered, brief diet history questionnaire (BDHQ) [14]. The BDHQ is a four-page structured questionnaire that enquires about the consumption frequency of a total of 56 foods and beverages that are commonly consumed in the general Japanese population. Dietary intakes, in terms of energy and selected nutrients, were estimated by applying an ad hoc computer algorithm to the 56 foods and beverages of the BDHQ and the Standard Tables of Food Composition in Japan [15].

### 2.5. Assessment of Sleep Duration and Sleep Timing

Sleep duration was assessed using the Japanese version of the Pittsburgh Sleep Quality Index (PSQI) [16,17]. The PSQI is a self-rated questionnaire that measures sleep difficulty retrospectively for a one-month period, with a global score ranging from 0 to 21. Higher PSQI scores indicate a lower quality of sleep. In the PSQI, the subjects reported bedtimes, sleep onset latency, and rise times. Using these data, we calculated the sleep duration by subtracting the sleep onset time from the rise time and the midpoint of sleep as the halfway point between sleep onset time and rise time to determine sleep timing [13].

### 2.6. Statistical Analysis

The average values of dietary intake and anthropometric variables were calculated. For energy adjustment, we used the percentages of total energy intakes (% energy) from macronutrients and alcohol and the intakes per 1000 kcal (/1000 kcal) for other nutrients and food. In this study, we evaluated energy (kcal/day), alcohol (% energy), protein (% energy), total fat (% energy), carbohydrate (% energy), cholesterol (mg/1000 kcal), sodium (mg/1000 kcal), potassium (mg/1000 kcal), calcium (mg/1000 kcal), magnesium (mg/1000 kcal), iron (mg/1000 kcal), zinc (mg/1000 kcal), vitamin A (µg/1000 kcal), vitamin D (µg/1000 kcal), vitamin E (mg/1000 kcal), thiamin (mg/1000 kcal), riboflavin (mg/1000 kcal), vitamin B6 (mg/1000 kcal), vitamin B12 (µg/1000 kcal), folate (µg/1000 kcal), and vitamin C (mg/1000 kcal), as well as rice (g/1000 kcal), noodles (g/1000 kcal), bread (g/1000 kcal), confections (g/1000 kcal), potatoes (g/1000 kcal), fat and oil (g/1000 kcal), fruits (g/1000 kcal), vegetables (g/1000 kcal), pulses (g/1000 kcal), fish and shell fish (g/1000 kcal), meat (g/1000 kcal), eggs (g/1000 kcal), and milk and milk products (g/1000 kcal).

The Student’s *t*-test was used to compare continuous variables, and the chi-square test was used to compare categorical variables between men and women. The multicolinearity effect was checked using VIF (Variance Inflation Factor) <10/tolerance tests >0.10. A Pearson’s correlation analysis was performed to examine the relationships between sleep duration and dietary intakes. A partial correlation procedure was used to examine the linear relationships between these variables after controlling for the effects of other variables (age, sleep timing). All analyses were performed using the statistical software SPSS version 15.0 (SPSS Japan, Inc., Tokyo, Japan). *p*-values < 0.05 were considered statistically significant.

## 3. Results

The mean age of all the subjects was 48.0 (10.3) years (mean (standard deviation)), and 54.1% of the subjects were male. The mean body mass index (BMI) was 22.4 (3.3). The characteristics and dietary intakes, stratified by sex, are shown in Table 1. We observed significant differences in age (*t*(1900) = 15.2, *p* = 0.001), sleep latency (*t*(1900) = 6.3, *p* = 0.001), and rise time (*t*(1900) = 0.66, *p* = 0.036) between men and women. We further observed significant differences in the percentages of current smokers (χ^2^(1) = 51.0, *p* = 0.001), subjects with a current exercise routine (χ^2^(1) = 47.1, *p* = 0.001), subjects who lived alone (χ^2^(1) = 43.0, *p* = 0.001), and occupational statuses (χ^2^(3) = 432.4, *p* = 0.001). A residual analysis revealed that among men, the percentage of full-time workers was significantly higher and the percentages of part-time workers and homemakers were significantly lower than among women.

Total energy and alcohol (% energy) intakes were significantly higher in male versus female subjects. On the other hand, male subjects had significantly lower intakes of protein (% energy), fat (% energy), and carbohydrates (% energy), as well as energy-adjusted (per 1000 kcal) nutrients other than sodium. Regarding food-group intakes (g/1000 kcal), those of bread, confections, potatoes, vegetables, pulses, fish and shell fish, meat, eggs, milk, and milk products were significantly higher among women than among men, whereas the reverse was true for rice and noodles.

Data regarding total energy; % energy from alcohol, protein, fat, and carbohydrates; and energy-adjusted (per 1000 kcal) nutrient and food-group intakes by sex are presented in Table 2 and Table 3, respectively. Regarding macronutrients, small but significant correlations were observed between sleep duration and the percentage of energy derived from protein, both with and without adjustments for the midpoint of sleep among men (*r* = 0.126, *p* < 0.05). The energy-adjusted intakes of sodium, vitamin D, and vitamin B12 correlated significantly with sleep duration in men after adjusting for the midpoint of sleep (sodium: *r* = 0.115, *p* < 0.01; vitamin D: *r* = 0.218, *p* < 0.01; vitamin B12: *r* = 0.192, *p* < 0.01).

Regarding food-group intakes, we observed significant correlations between sleep duration and the intakes of bread, pulses, and fish and shellfish among men, both with and without adjustment for the midpoint of sleep (bread: *r* = 0.092, *p* < 0.05; pulses: *r* = 0.141, *p* < 0.01; fish and shellfish: *r* = 0.183, *p* < 0.01).

In contrast, we observed no significant correlations between dietary intakes and sleep duration among women (Table 3).

## 4. Discussion

To the best of our knowledge, this is the first study to investigate the relationship between sleep duration and dietary intake of specific nutrients, while considering variations in sleep timing (i.e., the midpoint of sleep). In an earlier survey of a US population, energy intakes across sleep duration groups exhibited an inverse U-shaped distribution [8]. Another previous study also found an association of sleep deprivation with increased energy intake [7]. However, our results did not indicate a significant correlation between energy intakes and sleep duration. The reason for this discrepancy should be clarified in future studies.

We observed a significant sex-based difference in energy (kcal/day) intakes; specifically, male subjects had higher energy intakes. The observed sex-related differences in the intakes of several energy-adjusted nutrients, such as protein, calcium, and iron, are attributed to differences in total energy intake. After adjusting for the midpoint of sleep, we found that the intakes of specific dietary nutrients were correlated with sleep duration among men. The percentage of energy from protein and the energy-adjusted intakes of sodium, vitamin D, and vitamin B12 exhibited small but significant increases that correlated with sleep duration. In addition, the intakes of bread, pulses, and fish and shellfish were correlated with sleep duration, regardless of whether we adjusted for the midpoint of sleep. These results agree with those of a previous study that investigated the relationship between sleep duration and dietary intake in the NHANES; in that study, short sleepers reported lower intakes of protein, carbohydrates, dietary fiber, and total fats than did normal sleepers [8]. Another previous study of adolescents with short sleep durations observed decreased intakes of healthy foods such as vegetables, fruits, and fish, and increased intakes of unhealthy fast foods such as pizza, hamburgers, pasta dishes, and snack products [18]. Short sleep duration–induced changes in food preferences may be accompanied by changes in nutrient intakes, possibly consequent to changes in the secretion of appetite-related hormones such as leptin and ghrelin [5,6,19]. Previous studies also reported that total blood levels and circadian changes in cortisol, insulin, and thyroid-stimulating hormone levels were affected by a short sleep duration [20,21,22]. Additionally, a short sleep duration was found to enhance activity in brain reward and food-sensitive centers in response to unhealthy food stimuli [23]. A short sleep duration also led to extended hours of wakefulness, thus presenting additional opportunities for increased food intake [5]. Although these parameters were not evaluated objectively in the present study, they should be addressed in future studies. However, we observed no significant correlations between sleep duration and dietary intake among women in this study. We cannot clearly explain this sex-based difference. Previous studies either combined the data of men and women for analysis [8,12] or surveyed only women [13,24]. However, sex has been suggested as an important factor regarding food and nutrient intakes [25]. Our present study suggested a sex-based difference in the influence of sleep duration on dietary intake. Thus, the results of this study suggest a significant and independent association of sleep duration with dietary intakes of certain nutrients and foods in a Japanese adult male population after controlling for variations in the midpoint of sleep. Clock genes may influence the relationship between sleep timing and dietary intake. Circadian clocks, which are controlled by clock genes, regulate various biological rhythms, including sleep timing and the endocrine system [26]. In addition, mouse clock gene mutants exhibit increased alcohol intake [27]. Clock genes were also found to regulate metabolism [26]. In the meantime, periodic meal intake was found to be an important circadian clock entrainment signal in animals [28]. Furthermore, certain nutrients and food components, such as glucose, ethanol, caffeine, thiamine, and retinoic acid, can induce phase-shifts in circadian rhythms [29]. However, we could not identify if reverse causation occurred because this epidemiological study was cross-sectional. Further studies are required to understand the relationships between dietary intakes and circadian clocks in humans.

We should note several limitations of our study. First, information about both dietary nutrient intake and sleep duration was based on the participants’ self-reports. However, the participants might have overestimated their vegetable intake and/or underestimated their intakes of sweets and high-fat foods [14] during the preceding month. Still, the BDHQ was validated and used in several previous studies. Therefore, we used self-reporting methods to obtain data from our large sample. Further studies involving biomarkers and digital dietary records of nutrient intake are warranted. We also did not obtain information about the participants’ usage of caffeine, antidepressants, or other medications that could influence appetite and/or sleep. Furthermore, the sleep duration and midpoint of sleep were derived from the same questionnaire, despite the lack of a significant correlation between these two variables. The subjective sleep duration might also have been misclassified because of reporting errors, which warrants the use of an objective sleep measurement such as actigraphy. Second, the results of this study might have been affected by sampling bias, as health-conscious people may have been more likely to participate in this type of health survey. However, the mean nutrient and food intake values in this population were almost the same as those reported by similarly aged adults in the National Nutrition and Health Survey in Japan [30]. Therefore, the subjects of the present study may be representative of the general Japanese population, at least regarding the study variables. 

## 5. Conclusions

This study found that sleep duration was significantly and independently associated with the dietary intakes of certain nutrients and foods in a Japanese adult male population after controlling for variations in the midpoint of sleep.

## Figures and Tables

**Table 1:** Participant characteristics and dietary intakes, stratified by sex (*n* = 1902).

	All (*n* = 1902)		Male (*n* = 1029)		Female (*n* = 873)		*p*-Value
	Mean	SD	Mean	SD	Mean	SD	
Age, years	48.0	10.3	51.1	10.2	44.3	9.2	0.001
Body mass index, kg/m^2^	22.4	3.3	23.3	3.1	21.4	3.4	0.063
Bedtime, h:min	23:58	1:26	0:03	1:28	23:52	1:23	0.544
Sleep latency, min	19.4	19.0	16.9	15.0	22.4	22.6	0.001
Rise time, h:min	6:46	1:28	6:47	1:32	6:45	1:22	0.036
Midpoint of sleep, h:min	3:32	1:21	3:34	1:24	3:29	1:16	0.059
Sleep duration, h:min	6:26	1:05	6:23	1:04	6:30	1:07	0.311
Current smoking, %	22.0		28.3		14.7		0.001
Current exercise routine, %	27.7		34.2		20.1		0.001
Living alone, %	16.6		21.8		10.5		0.001
**Occupation**							
Full-time worker	61.8		77.9		42.6		0.001
Part-time worker	8.5		3.0		15.1		
Homemaker	11.6		0.0		25.3		
Unemployed	18.2		19.1		17.0		
Energy, kcal/day	1772.0	527.0	1922.5	521.6	1596.7	478.8	0.001
Alcohol, % energy	5.5	9.0	7.8	10.1	2.8	6.5	0.001
**Nutrients**							
Protein, % energy	14.5	2.9	13.9	2.7	15.3	3.0	0.001
Total fat, % energy	25.4	5.8	24.1	5.8	27.0	5.6	0.001
Carbohydrate, % energy	53.2	8.8	52.8	9.5	53.8	8.1	0.012
Cholesterol, mg/1000 kcal	196.0	75.0	184.3	75.5	210.4	73.1	0.001
Sodium, mg/1000 kcal	2313.0	491.0	2293.2	483.7	2337.1	499.8	0.052
Potassium, mg/1000 kcal	1336.0	404.0	1245.8	351.9	1442.8	435.0	0.001
Calcium, mg/1000 kcal	275.0	108.0	255.0	99.7	300.6	112.6	0.001
Magnesium, mg/1000 kcal	134.0	32.0	129.3	28.3	141.7	35.0	0.001
Iron, mg/1000 kcal	4.1	1.1	3.9	1.0	4.4	1.2	0.001
Zinc, mg/1000 kcal	4.2	0.7	4.1	0.7	4.4	0.7	0.001
Vitamin A, µg/1000 kcal	374.0	230.0	357.2	218.1	395.6	243.6	0.001
Vitamin D, µg/1000 kcal	6.5	4.3	6.1	3.9	6.9	4.6	0.001
Vitamin E, mg/1000 kcal	3.8	1.1	3.5	1.0	4.1	1.1	0.001
Thiamin, mg/1000 kcal	0.37	0.1	0.37	0.1	0.43	0.1	0.001
Riboflavin, mg/1000 kcal	0.69	0.2	0.66	0.2	0.73	0.2	0.001
Vitamin B6, mg/1000 kcal	0.65	0.2	0.62	0.2	0.69	0.2	0.001
Vitamin B12, µg/1000 kcal	4.6	2.4	4.5	2.3	4.8	2.6	0.006
Folate, µg/1000 kcal	176.0	68.0	164.0	58.7	190.9	75.6	0.001
Vitamin C, mg/1000 kcal	58.0	29.0	53.5	26.5	64.8	31.0	0.001
**Food Group (g/1000 kcal)**							
Rice	147.0	76.0	152.1	76.6	142.0	75.6	0.004
Noodles	44.0	30.0	49.2	33.1	38.2	26.3	0.001
Bread	49.0	28.0	43.5	25.8	55.6	30.9	0.001
Confections	27.0	21.0	22.5	17.9	33.5	24.1	0.001
Potatoes	18.0	17.0	19.2	18.4	25.7	21.8	0.001
Fat and oil	5.9	2.6	5.8	2.6	6.0	2.8	0.092
Fruits	62.0	58.0	61.4	59.2	63.9	57.2	0.362
Vegetables	134.0	82.0	116.3	66.2	155.0	95.1	0.001
Pulses	34.0	26.0	30.2	23.1	39.5	29.0	0.001
Fish and Shell fish	36.0	22.0	35.2	20.5	38.3	24.3	0.002
Meat	37.0	20.0	35.8	18.3	40.4	21.8	0.001
Eggs	20.0	14.0	19.8	14.7	21.8	14.7	0.003
Milk and milk products	68.0	61.0	63.5	61.4	73.4	61.2	0.001

SD: standard deviation.

**Table 2:** Correlation between sleep duration and dietary intakes among men (*n* = 1029).

	Mean	SD	^1^ *r*	^1^ β	^2^ *r*	^2^ β	^3^ *r*	^3^ β
Energy, kcal/day	1922.5	521.6	0.052	−0.05	0.134	−0.06	0.139	−0.06
Alcohol, % energy	7.8	10.1	0.024	0.02	0.150	0.02	0.156	0.02
**Nutrients**								
Protein, % energy	13.9	2.7	**0.076b**	0.08	**0.126b**	0.07	**0.126b**	0.07
Total fat, % energy	24.1	5.8	0.031	0.03	0.032	0.03	0.052	0.03
Carbohydrate, % energy	52.8	9.5	**0.068b**	−0.07	0.182	−0.06	0.183	−0.06
Cholesterol, mg/1000 kcal	184.3	75.5	0.058	0.06	0.113	0.05	0.114	0.05
Sodium, mg/1000 kcal	2293.2	483.7	**0.087a**	0.09	**0.093a**	0.09	**0.115a**	0.08
Potassium, mg/1000 kcal	1245.8	351.9	0.004	0.00	0.220	−0.02	0.224	−0.01
Calcium, mg/1000 kcal	255.0	99.7	0.011	0.01	0.212	0.00	0.218	0.00
Magnesium, mg/1000 kcal	129.3	28.3	0.030	0.03	0.226	0.02	0.230	0.02
Iron, mg/1000 kcal	3.9	1.0	0.045	0.05	0.180	0.04	0.182	0.04
Zinc, mg/1000 kcal	4.1	0.7	0.048	0.05	0.048	0.05	0.056	0.05
Vitamin A, µg/1000 kcal	357.2	218.1	0.014	0.01	0.074	0.01	0.096	0.02
Vitamin D, µg/1000 kcal	6.1	3.9	**0.088a**	0.09	**0.218a**	0.08	**0.218a**	0.08
Vitamin E, mg/1000 kcal	3.5	1.0	0.022	0.02	0.132	0.01	0.133	0.01
Thiamin, mg/1000 kcal	0.4	0.1	0.036	0.04	0.130	0.03	0.131	0.03
Riboflavin, mg/1000 kcal	0.7	0.2	0.026	0.03	0.187	0.02	0.193	0.02
Vitamin B6, mg/1000 kcal	0.6	0.2	0.058	0.06	0.208	0.05	0.213	0.05
Vitamin B12, µg/1000 kcal	4.5	2.3	**0.093a**	0.09	**0.192a**	0.08	**0.192a**	0.08
Folate, µg/1000 kcal	164.0	58.7	0.020	0.02	0.209	0.01	0.222	0.01
Vitamin C, mg/1000 kcal	53.5	26.5	0.007	0.01	0.242	−0.01	0.243	−0.01
**Food Group (g/1000 kcal)**								
Rice	152.1	76.6	0.025	−0.03	0.193	−0.01	0.197	−0.01
Noodles	49.2	33.1	0.038	0.04	0.114	0.04	0.143	0.04
Bread	43.5	25.8	0.056	−0.06	0.074	−0.06	**0.092b**	−0.06
Confections	22.5	17.9	0.054	−0.05	0.062	−0.06	0.083	−0.06
Potatoes	19.2	18.4	0.005	−0.01	0.044	−0.01	0.045	−0.01
Fat and oil	5.8	2.6	0.008	0.01	0.132	0.02	0.136	0.01
Fruits	61.4	59.2	0.005	−0.01	0.166	−0.02	0.167	−0.02
Vegetables	116.3	66.2	0.005	0.01	0.170	−0.01	0.178	0.00
Pulses	30.2	23.1	**0.086a**	0.09	**0.134a**	0.08	**0.141a**	0.08
Fish and Shell fish	35.2	20.5	**0.096a**	0.10	**0.183a**	0.09	**0.183a**	0.09
Meat	35.8	18.3	0.016	0.02	0.133	0.02	0.134	0.02
Eggs	19.8	14.7	0.046	0.05	0.075	0.04	0.081	0.05
Milk and milk products	63.5	61.4	0.031	−0.03	0.093	−0.04	0.099	−0.03

SD: standard deviation; *r*: Pearson’s correlation coefficient *r*; β: standardized coefficient β; ^1^: Univariate regression analysis; ^2^: Multivariate regression analysis (sleep duration, age); ^3^: Multivariate regression analysis (sleep duration, age, midpoint of sleep); a: *p* < 0.01, b: *p* < 0.05, number in bold showed significant variables.

**Table 3:** Correlation between sleep duration and dietary intakes among women (*n* = 873).

	Mean	SD	^1^ *r*	^1^ β	^2^ *r*	^2^ β	^3^ *r*	^3^ β
Energy, kcal/day	1596.7	478.8	0.002	0.00	0.097	0.01	0.109	0.01
Alcohol, % energy	2.8	6.5	0.018	−0.02	0.031	−0.02	0.068	−0.02
Nutrients								
Protein, % energy	15.3	3.0	0.019	0.02	0.147	0.03	0.160	0.03
Total fat, % energy	27.0	5.6	0.051	−0.05	0.064	−0.05	0.068	−0.05
Carbohydrate, % energy	53.8	8.1	0.035	0.04	0.056	0.03	0.057	0.03
Cholesterol, mg/1000 kcal	210.4	73.1	0.026	0.03	0.043	0.03	0.081	0.03
Sodium, mg/1000 kcal	2337.1	499.8	0.049	0.05	0.097	0.06	0.099	0.06
Potassium, mg/1000 kcal	1442.8	435.0	0.039	−0.04	0.217	−0.02	0.222	−0.02
Calcium, mg/1000 kcal	300.6	112.6	0.047	−0.05	0.204	−0.03	0.208	−0.03
Magnesium, mg/1000 kcal	141.7	35.0	0.014	−0.01	0.231	0.01	0.234	0.00
Iron, mg/1000 kcal	4.4	1.2	0.008	−0.01	0.157	0.01	0.161	0.00
Zinc, mg/1000 kcal	4.4	0.7	0.048	0.05	0.119	0.06	0.137	0.06
Vitamin A, µg/1000 kcal	395.6	243.6	0.008	−0.01	0.037	−0.01	0.038	−0.01
Vitamin D, µg/1000 kcal	6.9	4.6	0.017	0.02	0.140	0.03	0.145	0.03
Vitamin E, mg/1000 kcal	4.1	1.1	0.018	−0.02	0.133	−0.01	0.146	−0.01
Thiamin, mg/1000 kcal	0.4	0.1	0.013	−0.01	0.160	0.00	0.166	0.00
Riboflavin, mg/1000 kcal	0.7	0.2	0.050	−0.05	0.177	−0.04	0.178	−0.04
Vitamin B6, mg/1000 kcal	0.7	0.2	0.021	−0.02	0.159	−0.01	0.166	−0.01
Vitamin B12, µg/1000 kcal	4.8	2.6	0.013	−0.01	0.131	0.00	0.131	0.00
Folate, µg/1000 kcal	190.9	75.6	0.015	−0.02	0.166	0.00	0.170	0.00
Vitamin C, mg/1000 kcal	64.8	31.0	0.013	−0.01	0.186	0.00	0.187	0.00
Food group (g/1000 kcal)								
Rice	142.0	75.6	0.045	0.05	0.090	0.04	0.106	0.04
Noodles	38.2	26.3	0.014	0.01	0.089	0.01	0.137	0.01
Bread	55.6	30.9	0.043	−0.04	0.043	−0.04	0.044	−0.04
Confections	33.5	24.1	0.029	−0.03	0.069	−0.03	0.093	−0.03
Potatoes	25.7	21.8	0.038	−0.04	0.051	−0.04	0.068	−0.04
Fat and oil	6.0	2.8	0.024	−0.02	0.060	−0.03	0.060	−0.03
Fruits	63.9	57.2	0.008	0.01	0.135	0.02	0.137	0.02
Vegetables	155.0	95.1	0.034	−0.03	0.138	−0.02	0.141	−0.02
Pulses	39.5	29.0	0.006	0.01	0.121	0.02	0.122	0.02
Fish and Shell fish	38.3	24.3	0.018	0.02	0.129	0.03	0.129	0.03
Meat	40.4	21.8	0.002	0.00	0.039	−0.01	0.046	−0.01
Eggs	21.8	14.7	0.002	0.00	0.020	0.00	0.094	0.00
Milk and milk products	73.4	61.2	**0.066b**	−0.07	0.132	−0.06	0.132	−0.06

SD: standard deviation, *r*: Pearson’s correlation coefficient *r*; β: standardized coefficient β; ^1^: Univariate regression analysis; ^2^: Multivariate regression analysis (sleep duration, age); ^3^: Multivariate regression analysis (sleep duration, age, midpoint of sleep); a: *p* < 0.01, b: *p* < 0.05, number in bold showed significant variables.
